# Diagnostic reliability of serum active B12 (holo-transcobalamin) in true evaluation of vitamin B12 deficiency: Relevance in current perspective

**DOI:** 10.1186/s13104-022-06224-8

**Published:** 2022-10-22

**Authors:** Rinini Dastidar, Kunal Sikder

**Affiliations:** 1 Department of Biochemistry, Ramakrishna Mission Seva Pratishthan (RKMSP) hospital, 700026 Kolkata, West Bengal India; 2 Department of Biomedical Science and Technology, School of Biological Sciences, JIVAN, Ramakrishna Mission Vivekananda Educational and Research Institute (RKMVERI), 700026 Kolkata, West Bengal India

**Keywords:** Transcobalamin, Haptocorrin, Holo-transcobalamin, Vitamin B12 deficiency, Homocysteine, Diagnostic blind spot

## Abstract

**Objective:**

Measurement of total vitamin B12 (vit B12) concentration raised concerns over early detection of vit B12 deficiency due to its clinical unreliability. In this present article we aimed to assess the efficacy of holo-transcobolamin (active vit B12) for true evaluation of vit B12 deficiency.

**Methods:**

This retrospective study included 100 participants referred for vit B12 assay. Serum total vit B12, active vit B12 and homocysteine were estimated.

**Results:**

Our study showed 59% of the total participants with vit B12 deficiency (185 ± 64.62 pg/ml) and 18% with hyper-cobalaminemia (1666.9 ± 367.13 pg/ml) based on their total vit B12 concentrations. A comparative study on total vit B12 and active vit B12 was done which reflected a striking disparity in results. Active vit B12 reported 28.8% patients with vit B12 deficiency (19.8 ± 17.48 pg/ml) and only 16.6% patients with hyper-cobalaminemia (224.14 ± 10 pg/ml). Active vit B12 appeared to be more sensitive (82.35% vs 65%) and specific (46.6% vs. 43.8%) diagnostic marker compared to total vit B12. Pearson Correlation study indicated a strong positive correlation (r = 0.695 at *p* < 0.01) hence justified use of the two methods.

**Conclusion:**

We claim that active vit B12 is a much more reliable biomarker than total vit B12 for early diagnosis of vit B12 deficiency.

## Introduction

Vit B12 is an essential water-soluble micronutrient necessary for all cells of human body. Since endogenous synthesis does not occur in humans, they rely solely upon its proper dietary intake, processing, and delivery to the target tissues [1]. Dietary inadequacy and malabsorption of vit B12 have created a global disease burden including India [2, 3]. More often micronutrient deficiency is observed in older population [4]. Emerging evidence indicated a significant upsurge of vit B12 deficiency in infants, school going children [5, 6], adolescents, pregnant women and women of reproductive age [7, 8]. Vitamin D deficiency is perceived to prevent vit B12 absorption causing vit B12 deficiency and there is a significant positive correlation of vitamin D deficiency with vit B12 deficiency[9]. The current prevalence of vitamin D deficiency is 70–80% in school going children [10] and 90–95% in pregnant women[11] which might attribute to increased prevalence of vitamin B12 deficiency in them. Anemia caused by vit B12 deficiency is quite common in women of reproductive age in India which might lead to adverse perinatal outcome if timely intervention is not taken[12, 13]. A large spectrum of heterogeneous diseases[14] including irreversible neurological impairments[15], hematological disorders[16] and autoimmune disorders[17]are associated with vit B12 deficiency.Vit B12 deficiency has been linked with some tropical diseases in some studies but the potential role of vitamin B12 in the pathogenesis of the diseases is yet to be elucidated[18]. Growing trends of vegetarianism also imparts to the ascending curve of vit B12 deficiency in India[19]. There are some recent studies indicating the fat mass and obesity associated (FTO) gene polymorphism as one of the causal factors with vit B12 deficiency in obese persons who are considered to be the high candidates for type 2 diabetes [20]. Vitamin B12 deficiency is reported in individuals who are on metformin [21]and proton pump inhibitors for a prolonged period of time[22]. Laboratory investigation of serum vit B12 level is a routine practice for determining its deficiency. Raised serum methylmalonic acid (MMA) and homocysteine along with the reduced concentration of serum total vit B12 are the cornerstones in diagnostic evaluation of vit B12 deficiency [23], although the elevated concentrations of serum MMA and homocysteine are not always trustworthy in terms of specificity. Some diseases like renal impairment and folate/Vit B6 deficiency nonspecifically increase MMA [24] and homocysteine concentrations [25]. The unreliability of total vit B12 as diagnostic marker for the correct assessment of its deficiency has been reflected in several studies[26]. Low levels of serum vit B12 is not always accompanied with vit B12 deficiency, 15-40% of the B12 deficient patients are not truly deficient [27]. On the contrary normal levels of vit B12 with normal MMA/homocysteine cannot rule out the possibilities of having vit B12 deficiency [28]. Even asymptomatic patients with normal hematological profile can have low serum vit B12 concentration. Measurement of total transcobalamin (TC) sometimes leads to anomalies in test results [29]. It is a known fact that total vit B12 consists of 70–90% metabolically inert haptocorrin (HC) bound B12 (HC-B12-transcobalamin) and 6–20% biologically active transcobalamin II (active B12)[30]. HC-B12-transcobalamin estimation might be misleading, since HC concentration is reported to be decreased in pregnancy, aplastic anemia, and women with estrogen therapy [31]. On the other hand, some clinical conditions such as hypothyroidism, liver dysfunction and pernicious anemia which elevate HC concentration leading to falsely elevation of vit B12 level. Holo-TC appears to be an ideal marker in diagnosing true vit B12 status especially when total B12 assay shows subnormal or low results [32–34]. Cellular uptake of holo-TC bound fraction and its short half-life period makes it a more clinically meaningful marker than HC bound B12 for the early and reliable diagnosis of vit B12 deficiency. The aim of this retrospective study is to assess the performance of active vit B12 in comparison to total vit B12 for the correct diagnosis of true vitB12 status of the body.

## Methods

This is a retrospective study comprising data from a total 100 patients who visited Ramakrishna Mission Seva Pratishthan (RKMSP) hospital, a renowned general hospital for masses in the heart of the city, for a period of three months. The study was cleared by institutional ethics committee (IEC) of the same hospital, before collecting any data. Demographic characteristics and test results for total vit B12, active vit B12 and homocysteine were obtained from the medical records of Biochemistry Department of RKMSP hospital. Serum concentrations of total vit B12 was estimated on immunoassay analyzer (Cobas6000, Roche Diagnostics, Germany) by electro chemiluminescence method, active vit B12 was determined on auto analyzer (Abbot, Architect, Germany) by micro particle enzyme assay method and homocysteine was measured on biochemistry analyzer (Virtos 4600, Ortho Clinical Diagnostics, USA) by spectrophotometric method. The patients were divided into three groups based on their total vit B12 concentration. Group I: Elevated (Total vit B12 > 1000 pg ml) Group II: Sufficient (Total vit B12 = 300–946 pg/ml) and group III: Deficient (Total vit B12 < 300 pg/ml). Results were statistically compared by mean and standard deviation (Mean ± SD) with statistical software Graph pad prism 9.0. Pearson Correlation test was carried out to assess the correlation between total and active vit B12 by correlation coefficient. The results were evaluated to ascertain agreement or disagreement between total and active vit B12 test results for correct diagnosis of vit B12 deficiency, hyper-cobalaminemia and vit B12 adequacy in the study participants.The threshold values for total vit B12, active vit B12 and homocysteine were determined by their reference range cited in the kit literatures. Reference ranges are as follows:

Active vit B12: Deficient:<34 pg/ml, Sufficient:34–223 pg/ml, High:>223 pg/ml.

Total vit B12: Deficient:<300 pg/ml, Sufficient:300–946 pg/ml, High:>946 pg/ml.

Homocysteine: Deficient:<4 µmol/L, Sufficient:4–15 µmol/L, High:>15 µmol/L.

## Results

There were 55% males with a mean age 54.43 ± 11.09 years and 45% females with a mean age 51.11 ± 9.48 years in this study. 59% of the total participants showed vit B12 deficiency (185 ± 64.62 pg/ml), 18% showed hyper-cobalaminemia (1666.9 ± 367.13 pg/ml) and 23% showed vit B12 sufficiency (492.30 ± 198.68 pg/ml) based on their total vit B12 concentrations. On the contrary, active vit B12 concentration demonstrated only 17% patients were deficient (19.81 ± 7.48 pg/ml), 3%with hyper-cobalaminemia (224.14 ± 10pg/ml) and 80% with sufficient vit B12 level (107.70 ± 56.64 pg/ml). Among all the subjects, 24 reported folate deficiencies (3.23 ± 1.04 ng/ml) whereas folate concentration was sufficient (10.77 ± 4.98 ng/ml) in 76% of the total participants. Hyper-homocysteinemia (24.06 ± 11.77 µmol/L) and normo-homocysteinemia (11.87 ± 1.83 µmol/L) were observed in 42% and 58% of the study group participants respectively (Table 1).

**Table 1:** Demographic & Biochemical characteristics of the study participants

Parameters	n	(%)	Mean ± SD
Age (M)	55	55	54.43 ± 11.09 years
Age (F)	45	45	51.11 ± 9.48 years
Total B12 (D)	59	59	185.62 ± 64.62 pg/ml
Total B12 (H)	18	18	1666.90 ± 367.13 pg/ml
Total B12 (S)	23	23	492.30 ± 198.68 pg/ml
Active B12 (D)	17	17	19.81 ± 7.48 pg/ml
Active B12(H)	3	3	224.14 ± 10 pg/ml
Active B12 (S)	80	80	107.70 ± 56.64 pg/ml
Folate (D)	24	24	3.23 ± 1.04 ng/ml
Folate (S)	76	76	10.77 ± 4.98 ng/ml
Homocysteine (H)	42	42	24.06 ± 11.77 µmol/L
Homocysteine (S)	58	58	11.87 ± 1.83 µmol/L

M = male, F = female, D = deficient, H = high, S = sufficient, n = number of participants for each parameter

**Fig. 1:**
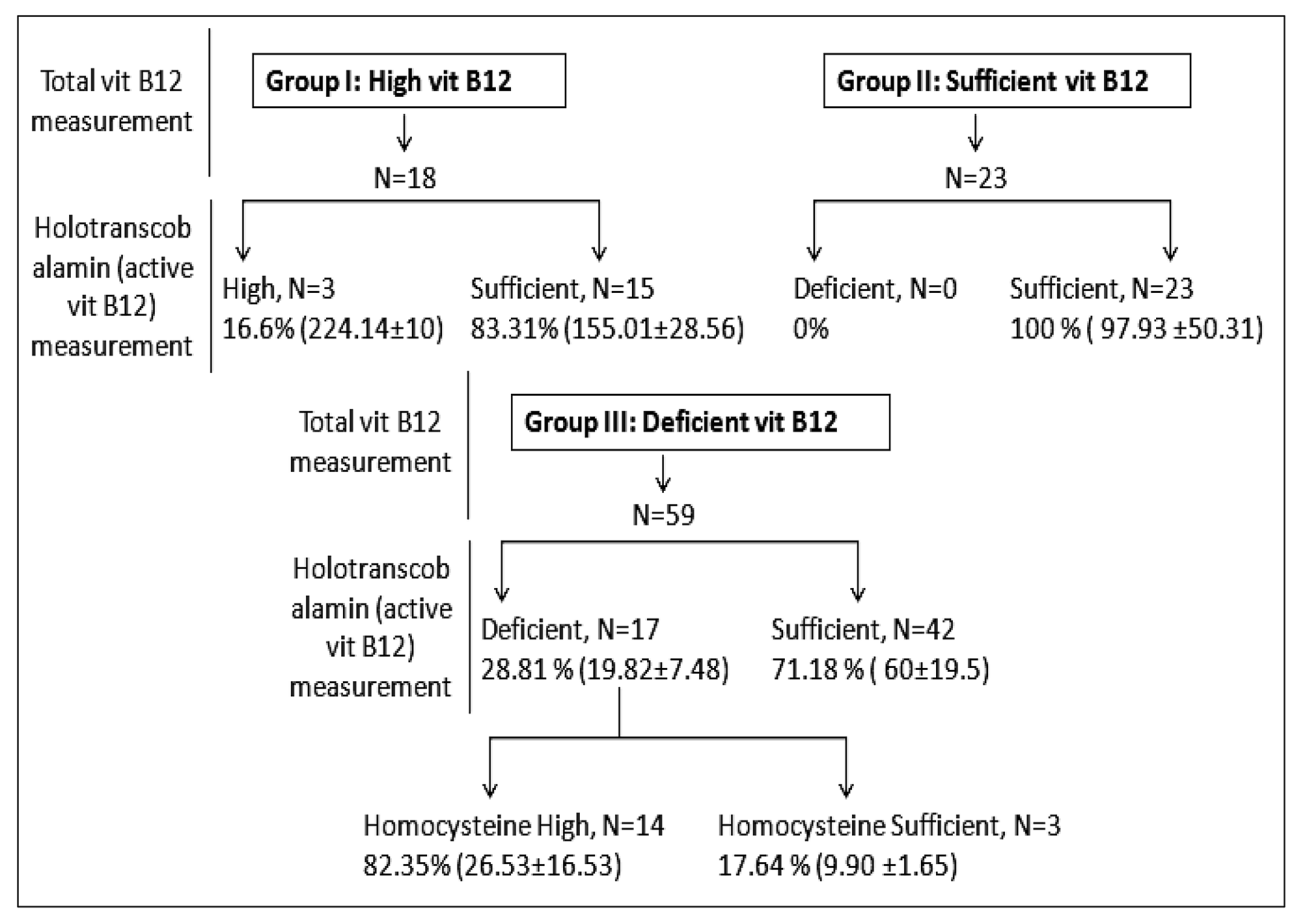
Active B12 concentration in group I (H: High), group II (S: Sufficient) and group III (D: Deficient) participants.

18% (N = 18), 23% (N = 23) and 59% (N = 59) of the total participants were included in group I or high vit B12 group (1666.9 ± 377.7 pg/ml), group II or sufficient vit B12 group (492.3 ± 198.6 pg/ml) and group III or deficient vit B12 group (185 ± 64.6 pg/ml) respectively (Fig. 1). Serum active vit B12 concentrations were measured in each group and results were compared with total vit B12 for assessing hyper-cobalaminemia, vit B12 sufficiency and deficiency (Fig. 1). 83.3% (N = 15 out of 18) participants in group I showed hyper-cobalaminemia with raised total vit B12 concentration (1600 ± 367.59 pg/ml) but their active vit B12 concentration lied (155.01 ± 28.56 pg/ml) within reference range (34–223 pg/ml). Only 3 participants out of 18 (16.6%) showed combined hyper-cobalaminemia with both elevated holo-TC (224.14 ± 10 pg/ml) and total vit B12 concentration (> 2000 pg/ml) in this group. Serum holo-TC (97.93 ± 50.31pg/ml) and total vit B12 (492.3 ± 198.6 pg/ml) test results in group II participants were in complete agreement with each other indicating they had sufficient levels of both total and active vit B12. A shocking disparity in test results was observed in vit B12 deficient group (Group III).71.18% (42 participants out of 59) here had sufficient holo-TC (60 ± 19.5 pg/ml) but reduced total vit B12 (216.38 ± 46.49 pg/ml). Combined vit B12 deficiency with reduced concentrations of both total vit B12 (109 ± 31.31pg/ml) and active vit B12 (19.82 ± 7.49 pg/ml) was observed in only 28.81% (17 participants out of 59) in the vit B12 deficient group. Hyper-homocysteinemia (26.53 ± 16.53µmol/L) was demonstrated in 82.35% (14 participantsout of 17) with combined vit B12 deficiency, whereas normo-homocysteinemia (9.90 ± 1.65 µmol/L) was observed in 17.6% participants (3 out of 17) in group III. True B12 deficiency was further corroborated with hyper-homocysteinemia (26.53 + 16.53 µmol/L) in the patients (Fig. 1)

**Fig. 2:**
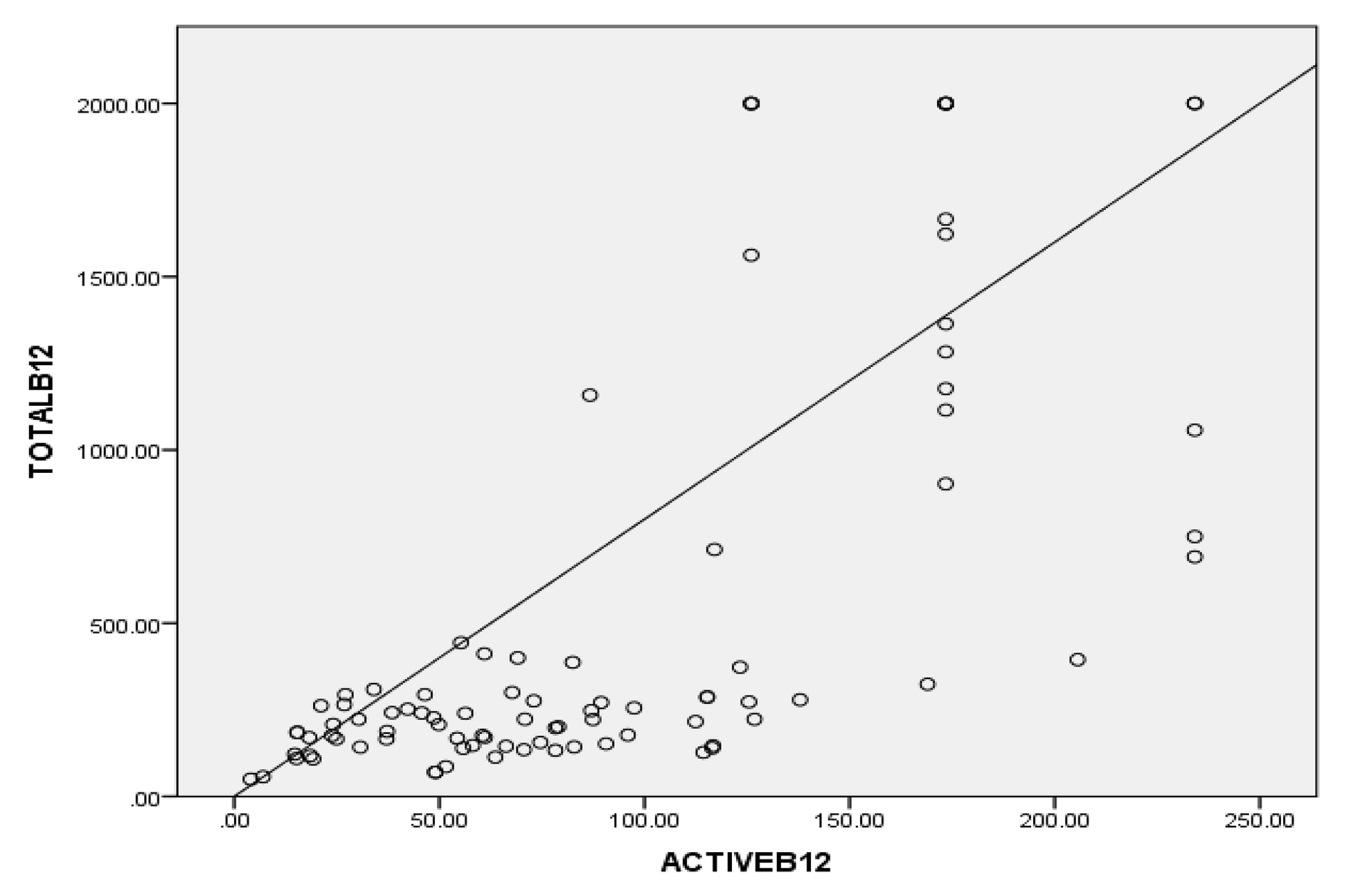
Scatter plot between total B12 and active B12 showed a significant positive correlation at 0.01

Concordance study with homocysteine showed high sensitivity of active vit B12 than total vit B12 (82.35% vs. 65%) but moderately high specificity was observed (46.6%vs43.8%) between the two. Pearson Correlation study of total vit B12 with active vit B12 indicated a strong positive correlation (r = 0.695, *p* < 0.01) between total B12 and active B12 (Fig. 2), thereby justifying the comparison of two methods for similar biochemical assessments.

## Discussion

Reduced or sub normal total vit B12 concentration does not always clinically correlate with vit B12 deficiency [35]. On the other hand, subclinical vit B12 deficiencies sometimes not reflected in reduced total vit B12 concentration, it remains borderline low or within normal reference range [36]. Active B12 or holo-TC appears as a rescue to override the clinical dilemma associated with total vit B12 testing. It has been claimed as a more reliable diagnostic marker for early detection of vit B12 deficiency due to its speedy cellular uptake and its short half-life period as compared to total vit B12. The other important attribute of active vit B12 which makes it superior to total vit B12 in terms of diagnostic accuracy is that malabsorption of intrinsic factor does not reduce active vit B12 concentration. Our study is also in compliance with numerous studies indicating holo-TC as diagnostically more accurate marker in comparison to total vit B12 due to its high sensitivity and specificity over total vit B12 [37, 38].

In this study we did a comparison between total and active vit B12 test results in various groups. Group II (B12 sufficient group) participants were in compliance with both vit B12 measurements, total and active respectively. But total B12 test results in group I and group III were not in alliance with their active vit B12 test results. Clinical history revealed only 16.6% (3 participant sout of 18) having clinical history of neuropathy were on vit B12 supplementation in group I and they showed raised concentrations of both active and total B12. Among the other patients with elevated vit B12 concentration (groupI) 6 had CKD (not on supplementation), 5 had viral hepatitis and 4 patients developed malignancy induced anemia. Hyper-cobalaminemia in group I participants without history of supplementation might be attributed totheir elevated serum HC concentration which has been reported to be elevated in several diseases including kidney disease, chronic liver disease, hematological malignancies [39–41]. Two possible hypotheses are there for such elevation, firstly the leakage of holo-haptocorrin in the circulation by the damaged hepatocytes in patients with acute hepatitis.Secondly, reduced cellular clearance of holo-haptocorrin by the injured hepatic cells causing false elevation of total vit B12 concentration. We also observed a striking difference intotal and active vit B12 test results in vit B12 deficient group (Group III). 71.18% in group III showed vit B12 deficiency with reduced total vit B12 concentration (216.38 ± 46.49 pg/ml) without showing any signs or symptoms of clinical vit B12 deficiency.They were tested for active vit B12 which was observed to be in sufficient concentration (60 ± 19.5 pg/ml) in the participants. Numerous studies reported that reduced total vit B12 concentrations do not always accompany with clinical B12 deficiency [42]. Only 28.81% patients in group III who had combined vit B12 deficiency with reduced total and active vit B12 concentrations showed clinical manifestations of vit B12 deficiency. Vit B12 deficiency was further corroborated with hyper-homocysteinemia (26.53 ± 16.53 µmol/L) in 82.35% (14 participants out of 17) with combined vit B12 deficiency in group III. But on a practical stand point active vit B12 has still not gained much attention in a country like India due to its high cost and limited availability despite having stupendous diagnostic accuracy. Most of the laboratories in India still depend on total vit B12 for detecting vit B12 deficiency which produces confusing test results, sometimes indicates vit B12 deficiency in apparently healthy normal individuals without showing clinical manifestations [43]. Likewise, sometimes yields false normal test values [44] in the patients with clinical vit B12 deficiency. Hence clinical evaluation of the patients along with vit B12 diagnostic reports should be considered for accurate diagnosis of vit B12 deficiency.

## Conclusion

We believe our study bears a significant relevance to the clinicians, patients, and all other stake holders as it will enable to create much awareness of adopting active vitamin B12 test for accurate diagnosis of vit B12 deficiency.

## Limitations

The sample number in this study was limited for any validation as was the population type. The future study must be applied to a wide range of population keeping in mind any racial and ethnic variation with a larger sample size. MMAis one of the most important biomarkers for the confirmation of vitamin B12 deficiency which we could not afford to test due to financial restraints.

## Data Availability

The datasets used during the current study available from the corresponding author on reasonable request.

## Abbreviations

Vit B12: Vitamin B12
MMA: Methylmalonic acid
HC: Haptocorrin
TC: Transcobalamin
